# A31 DYSPLASIA IN COLONIC POLYPS: PREPARING FOR A RESECT AND DISCARD STRATEGY IN CANADA

**DOI:** 10.1093/jcag/gwad061.031

**Published:** 2024-02-14

**Authors:** V Patel, R Bechara, M S Rai

**Affiliations:** Medicine, Queen's University, Kingston, ON, Canada; Medicine, Queen's University, Kingston, ON, Canada; Medicine, Queen's University, Kingston, ON, Canada

## Abstract

**Background:**

While diminutive colorectal polyps have a negligible cancer risk, current management involves resecting and submitting all polyps for histological assessment. This is a substantial burden and cost to the healthcare system. The European Society of Gastrointestinal Endoscopy (ESGE) has recommended a “resect-and-discard strategy” without histological evaluation as an acceptable strategy when high-confidence endoscopic characterization of colorectal polyps is achieved. However, a resect and discard strategy has not been adopted yet in Canada.

**Aims:**

The objective of this study was to determine the prevalence and characteristics of polyps removed at our center based on size.

**Methods:**

We retrospectively reviewed colonoscopies and pathology reports at Kingston Health Sciences Center from January to December 2020. Based on the pathology report, polyps were classified as diminutive (1-5mm), small (6-9mm) or large (ampersand:003E or = 10mm). We recorded histology and the presence of high-grade dysplasia (HGD) or cancer.

**Results:**

Out of 2218 colonoscopies, 2945 polyps were removed. 1703 (57.8%) polyps were diminutive with only two (0.1%) having focal HGD and none having cancer. 699 (23.7%) polyps were classified as small with three (0.4%) having HGD and none having cancer. The large polyp group had 543 (18.4%) polyps, of which 87 (16%) showed HGD and 15 (2.8%) exhibited cancer. The specific histologic findings are shown in Table 1. Endoscopy reports specifically mentioned concern of dysplasia in one out of the five polyps with HGD in the diminutive and small groups.

**Conclusions:**

As expected, most polyps were either diminutive or small (81.5%). Neither of these groups had cancer and only 5 had HGD. Adopting a resect and discard strategy at our center for diminutive polyps has the potential for significant cost savings with negligible risk of missing a high-risk polyp. The next steps would involve assessing optical diagnosis sensitivity and specificity for diminutive and small polyps.

Histological characterization of polyps within each size range

**Funding Agencies:**

None

**Table:** 

Histology	Diminutive (1-5mm)	Small (6-9mm)	Large (≥10mm)
Tubular adenoma	1212 (71.2%)	459 (65.7%)	274 (50.5%)
Tubular adenoma with HGD	1 (0.1%)	2 (0.3%)	32 (5.9%)
Hyperplastic	347 (20.4%)	100 (14.3%)	29 (5.3%)
Sessile Serrated	107 (6.3%)	107 (15.3%)	73 (13.4%)
Sessile Serrated with HGD	1 (0.1%)	0	4 (0.7%)
Tubulovillous	19 (1.1%)	25 (3.6%)	77 (14.2%)
Tubulovillous with HGD	0	1 (0.1%)	36 (6.6%)
Inflammatory	16 (0.9%)	5 (0.7%)	3 (0.6%)
Adenocarcinoma	0	0	15 (2.8%)
Total	1703	699	543

